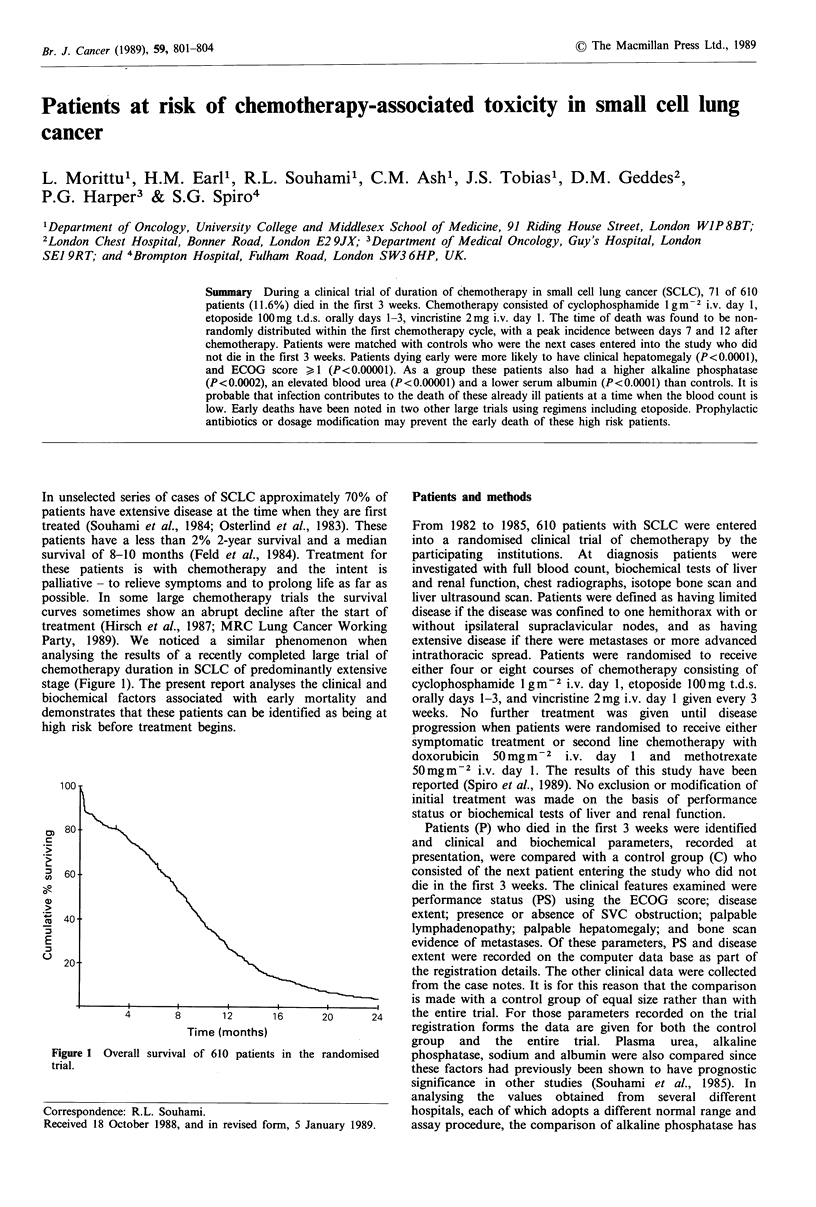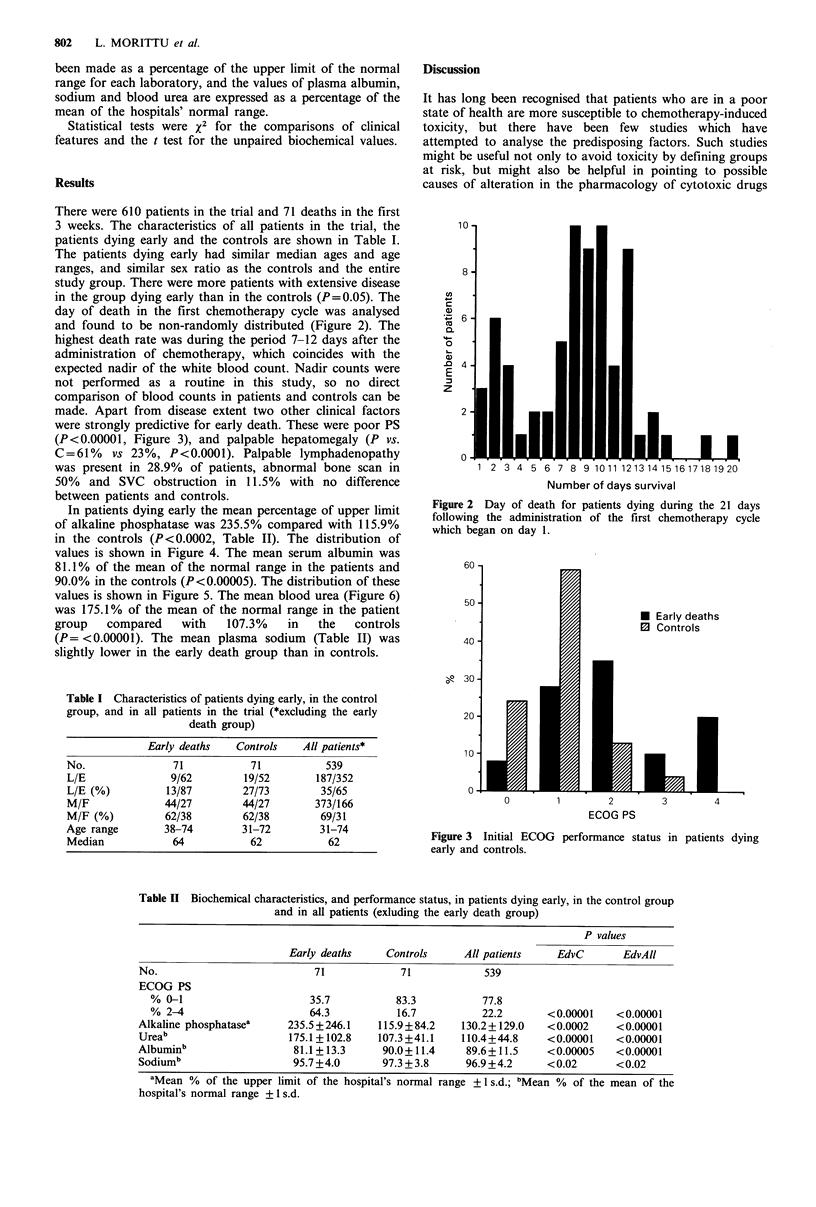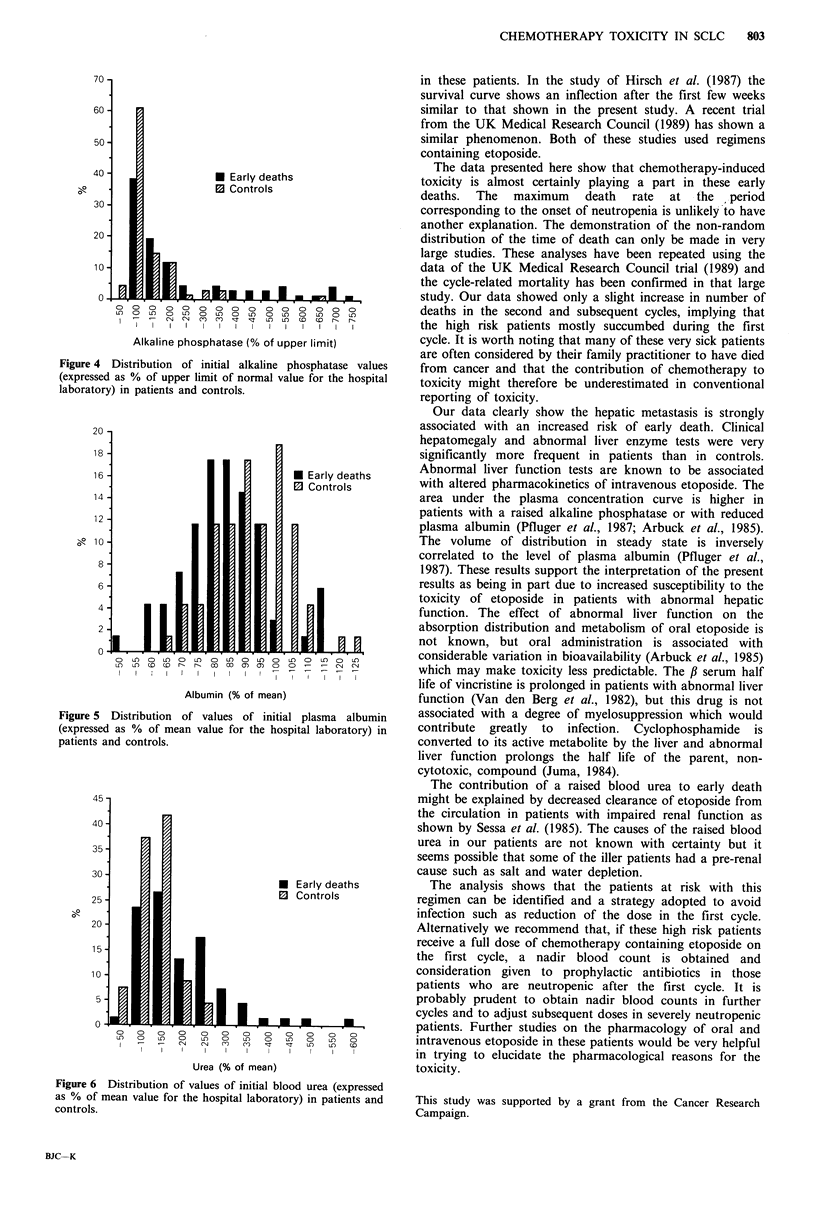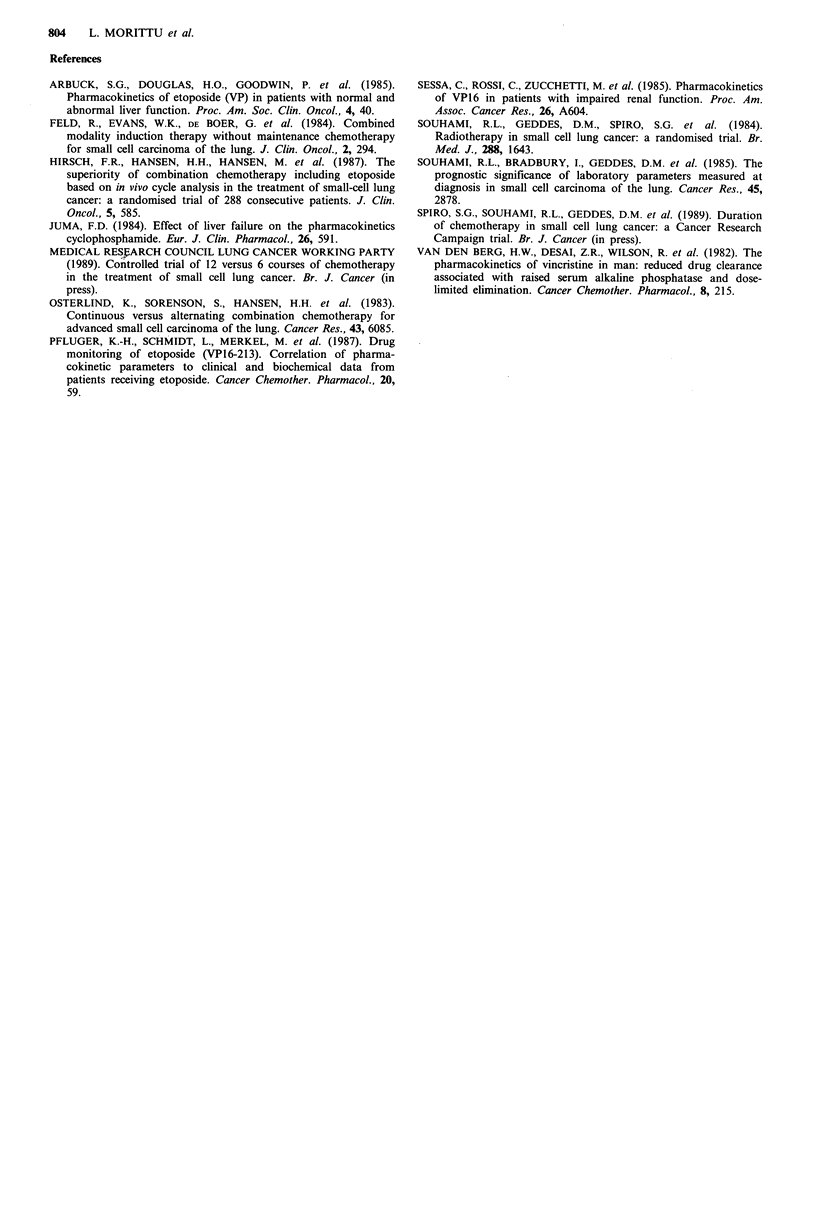# Patients at risk of chemotherapy-associated toxicity in small cell lung cancer.

**DOI:** 10.1038/bjc.1989.167

**Published:** 1989-05

**Authors:** L. Morittu, H. M. Earl, R. L. Souhami, C. M. Ash, J. S. Tobias, D. M. Geddes, P. G. Harper, S. G. Spiro

**Affiliations:** Department of Oncology, University College and Middlesex School of Medicine, London, UK.

## Abstract

During a clinical trial of duration of chemotherapy in small cell lung cancer (SCLC), 71 of 610 patients (11.6%) died in the first 3 weeks. Chemotherapy consisted of cyclophosphamide 1 g m-2 i.v. day 1, etoposide 100 mg t.d.s. orally days 1-3, vincristine 2 mg i.v. day 1. The time of death was found to be nonrandomly distributed within the first chemotherapy cycle, with a peak incidence between days 7 and 12 after chemotherapy. Patients were matched with controls who were the next cases entered into the study who did not die in the first 3 weeks. Patients dying early were more likely to have clinical hepatomegaly (P less than 0.0001), and ECOG score greater than or equal to 1 (P less than 0.00001). As a group these patients also had a higher alkaline phosphatase (P less than 0.0002), an elevated blood urea (P less than 0.00001) and a lower serum albumin (P less than 0.0001) than controls. It is probable that infection contributes to the death of these already ill patients at a time when the blood count is low. Early deaths have been noted in two other large trials using regimens including etoposide. Prophylactic antibiotics or dosage modification may prevent the early death of these high risk patients.


*Br. J. Cancer* (1989), **59**, 801–804 © The Macmillan Press Ltd., 1989

### Patients at risk of chemotherapy-associated toxicity in small cell lung cancer

L. Morittu[1], H.M. Earl[1], R.L. Souhami[1], C.M. Ash[1], J.S. Tobias[1], D.M. Geddes[2], P.G. Harper[3] & S.G. Spiro[4]

[1]*Department of Oncology, University College and Middlesex School of Medicine, 91 Riding House Street, London W1P 8BT;* [2]*London Chest Hospital, Bonner Road, London E2 9JX;* [3]*Department of Medical Oncology, Guy's Hospital, London SE1 9RT; and* [4]*Brompton Hospital, Fulham Road, London SW3 6HP, UK.*

**Summary** During a clinical trial of duration of chemotherapy in small cell lung cancer (SCLC), 71 of 610 patients (11.6%) died in the first 3 weeks. Chemotherapy consisted of cyclophosphamide $1\,g\,m^{-2}$ i.v. day 1, etoposide 100 mg t.d.s. orally days 1–3, vincristine 2 mg i.v. day 1. The time of death was found to be non-randomly distributed within the first chemotherapy cycle, with a peak incidence between days 7 and 12 after chemotherapy. Patients were matched with controls who were the next cases entered into the study who did not die in the first 3 weeks. Patients dying early were more likely to have clinical hepatomegaly ($P<0.0001$), and ECOG score $\geqslant 1$ ($P<0.00001$). As a group these patients also had a higher alkaline phosphatase ($P<0.0002$), an elevated blood urea ($P<0.00001$) and a lower serum albumin ($P<0.0001$) than controls. It is probable that infection contributes to the death of these already ill patients at a time when the blood count is low. Early deaths have been noted in two other large trials using regimens including etoposide. Prophylactic antibiotics or dosage modification may prevent the early death of these high risk patients.

In unselected series of cases of SCLC approximately 70% of patients have extensive disease at the time when they are first treated (Souhami *et al.*, 1984; Osterlind *et al.*, 1983). These patients have a less than 2% 2-year survival and a median survival of 8–10 months (Feld *et al.*, 1984). Treatment for these patients is with chemotherapy and the intent is palliative – to relieve symptoms and to prolong life as far as possible. In some large chemotherapy trials the survival curves sometimes show an abrupt decline after the start of treatment (Hirsch *et al.*, 1987; MRC Lung Cancer Working Party, 1989). We noticed a similar phenomenon when analysing the results of a recently completed large trial of chemotherapy duration in SCLC of predominantly extensive stage (Figure 1). The present report analyses the clinical and biochemical factors associated with early mortality and demonstrates that these patients can be identified as being at high risk before treatment begins.

**Figure 1** Overall survival of 610 patients in the randomised trial.

Correspondence: R.L. Souhami.
Received 18 October 1988, and in revised form, 5 January 1989.

### Patients and methods

From 1982 to 1985, 610 patients with SCLC were entered into a randomised clinical trial of chemotherapy by the participating institutions. At diagnosis patients were investigated with full blood count, biochemical tests of liver and renal function, chest radiographs, isotope bone scan and liver ultrasound scan. Patients were defined as having limited disease if the disease was confined to one hemithorax with or without ipsilateral supraclavicular nodes, and as having extensive disease if there were metastases or more advanced intrathoracic spread. Patients were randomised to receive either four or eight courses of chemotherapy consisting of cyclophosphamide $1\,g\,m^{-2}$ i.v. day 1, etoposide 100 mg t.d.s. orally days 1–3, and vincristine 2 mg i.v. day 1 given every 3 weeks. No further treatment was given until disease progression when patients were randomised to receive either symptomatic treatment or second line chemotherapy with doxorubicin $50\,mg\,m^{-2}$ i.v. day 1 and methotrexate $50\,mg\,m^{-2}$ i.v. day 1. The results of this study have been reported (Spiro *et al.*, 1989). No exclusion or modification of initial treatment was made on the basis of performance status or biochemical tests of liver and renal function.

Patients (P) who died in the first 3 weeks were identified and clinical and biochemical parameters, recorded at presentation, were compared with a control group (C) who consisted of the next patient entering the study who did not die in the first 3 weeks. The clinical features examined were performance status (PS) using the ECOG score; disease extent; presence or absence of SVC obstruction; palpable lymphadenopathy; palpable hepatomegaly; and bone scan evidence of metastases. Of these parameters, PS and disease extent were recorded on the computer data base as part of the registration details. The other clinical data were collected from the case notes. It is for this reason that the comparison is made with a control group of equal size rather than with the entire trial. For those parameters recorded on the trial registration forms the data are given for both the control group and the entire trial. Plasma urea, alkaline phosphatase, sodium and albumin were also compared since these factors had previously been shown to have prognostic significance in other studies (Souhami *et al.*, 1985). In analysing the values obtained from several different hospitals, each of which adopts a different normal range and assay procedure, the comparison of alkaline phosphatase has been made as a percentage of the upper limit of the normal range for each laboratory, and the values of plasma albumin, sodium and blood urea are expressed as a percentage of the mean of the hospitals' normal range.

Statistical tests were $\chi^2$ for the comparisons of clinical features and the $t$ test for the unpaired biochemical values.

### Results

There were 610 patients in the trial and 71 deaths in the first 3 weeks. The characteristics of all patients in the trial, the patients dying early and the controls are shown in Table I. The patients dying early had similar median ages and age ranges, and similar sex ratio as the controls and the entire study group. There were more patients with extensive disease in the group dying early than in the controls ($P=0.05$). The day of death in the first chemotherapy cycle was analysed and found to be non-randomly distributed (Figure 2). The highest death rate was during the period 7–12 days after the administration of chemotherapy, which coincides with the expected nadir of the white blood count. Nadir counts were not performed as a routine in this study, so no direct comparison of blood counts in patients and controls can be made. Apart from disease extent two other clinical factors were strongly predictive for early death. These were poor PS ($P<0.00001$, Figure 3), and palpable hepatomegaly (*P vs.* C=61% *vs* 23%, $P<0.0001$). Palpable lymphadenopathy was present in 28.9% of patients, abnormal bone scan in 50% and SVC obstruction in 11.5% with no difference between patients and controls.

In patients dying early the mean percentage of upper limit of alkaline phosphatase was 235.5% compared with 115.9% in the controls ($P<0.0002$, Table II). The distribution of values is shown in Figure 4. The mean serum albumin was 81.1% of the mean of the normal range in the patients and 90.0% in the controls ($P<0.00005$). The distribution of these values is shown in Figure 5. The mean blood urea (Figure 6) was 175.1% of the mean of the normal range in the patient group compared with 107.3% in the controls ($P=<0.00001$). The mean plasma sodium (Table II) was slightly lower in the early death group than in controls.

**Table I** Characteristics of patients dying early, in the control group, and in all patients in the trial (*excluding the early death group)

| | *Early deaths* | *Controls* | *All patients** |
|---|---|---|---|
| No. | 71 | 71 | 539 |
| L/E | 9/62 | 19/52 | 187/352 |
| L/E (%) | 13/87 | 27/73 | 35/65 |
| M/F | 44/27 | 44/27 | 373/166 |
| M/F (%) | 62/38 | 62/38 | 69/31 |
| Age range | 38–74 | 31–72 | 31–74 |
| Median | 64 | 62 | 62 |

### Discussion

It has long been recognised that patients who are in a poor state of health are more susceptible to chemotherapy-induced toxicity, but there have been few studies which have attempted to analyse the predisposing factors. Such studies might be useful not only to avoid toxicity by defining groups at risk, but might also be helpful in pointing to possible causes of alteration in the pharmacology of cytotoxic drugs

**Figure 2** Day of death for patients dying during the 21 days following the administration of the first chemotherapy cycle which began on day 1.

**Figure 3** Initial ECOG performance status in patients dying early and controls.

**Table II** Biochemical characteristics, and performance status, in patients dying early, in the control group and in all patients (exluding the early death group)

| | | | | *P values* | |
|---|---|---|---|---|---|
| | *Early deaths* | *Controls* | *All patients* | *EdvC* | *EdvAll* |
| No. | 71 | 71 | 539 | | |
| ECOG PS | | | | | |
| % 0–1 | 35.7 | 83.3 | 77.8 | | |
| % 2–4 | 64.3 | 16.7 | 22.2 | <0.00001 | <0.00001 |
| Alkaline phosphatase[a] | 235.5±246.1 | 115.9±84.2 | 130.2±129.0 | <0.0002 | <0.00001 |
| Urea[b] | 175.1±102.8 | 107.3±41.1 | 110.4±44.8 | <0.00001 | <0.00001 |
| Albumin[b] | 81.1±13.3 | 90.0±11.4 | 89.6±11.5 | <0.00005 | <0.00001 |
| Sodium[b] | 95.7±4.0 | 97.3±3.8 | 96.9±4.2 | <0.02 | <0.02 |

[a]Mean % of the upper limit of the hospital's normal range ±1 s.d.; [b]Mean % of the mean of the hospital's normal range ±1 s.d.

**Figure 4** Distribution of initial alkaline phosphatase values (expressed as % of upper limit of normal value for the hospital laboratory) in patients and controls.

**Figure 5** Distribution of values of initial plasma albumin (expressed as % of mean value for the hospital laboratory) in patients and controls.

**Figure 6** Distribution of values of initial blood urea (expressed as % of mean value for the hospital laboratory) in patients and controls.

in these patients. In the study of Hirsch *et al.* (1987) the survival curve shows an inflection after the first few weeks similar to that shown in the present study. A recent trial from the UK Medical Research Council (1989) has shown a similar phenomenon. Both of these studies used regimens containing etoposide.

The data presented here show that chemotherapy-induced toxicity is almost certainly playing a part in these early deaths. The maximum death rate at the period corresponding to the onset of neutropenia is unlikely to have another explanation. The demonstration of the non-random distribution of the time of death can only be made in very large studies. These analyses have been repeated using the data of the UK Medical Research Council trial (1989) and the cycle-related mortality has been confirmed in that large study. Our data showed only a slight increase in number of deaths in the second and subsequent cycles, implying that the high risk patients mostly succumbed during the first cycle. It is worth noting that many of these very sick patients are often considered by their family practitioner to have died from cancer and that the contribution of chemotherapy to toxicity might therefore be underestimated in conventional reporting of toxicity.

Our data clearly show the hepatic metastasis is strongly associated with an increased risk of early death. Clinical hepatomegaly and abnormal liver enzyme tests were very significantly more frequent in patients than in controls. Abnormal liver function tests are known to be associated with altered pharmacokinetics of intravenous etoposide. The area under the plasma concentration curve is higher in patients with a raised alkaline phosphatase or with reduced plasma albumin (Pfluger *et al.*, 1987; Arbuck *et al.*, 1985). The volume of distribution in steady state is inversely correlated to the level of plasma albumin (Pfluger *et al.*, 1987). These results support the interpretation of the present results as being in part due to increased susceptibility to the toxicity of etoposide in patients with abnormal hepatic function. The effect of abnormal liver function on the absorption distribution and metabolism of oral etoposide is not known, but oral administration is associated with considerable variation in bioavailability (Arbuck *et al.*, 1985) which may make toxicity less predictable. The $\beta$ serum half life of vincristine is prolonged in patients with abnormal liver function (Van den Berg *et al.*, 1982), but this drug is not associated with a degree of myelosuppression which would contribute greatly to infection. Cyclophosphamide is converted to its active metabolite by the liver and abnormal liver function prolongs the half life of the parent, non-cytotoxic, compound (Juma, 1984).

The contribution of a raised blood urea to early death might be explained by decreased clearance of etoposide from the circulation in patients with impaired renal function as shown by Sessa *et al.* (1985). The causes of the raised blood urea in our patients are not known with certainty but it seems possible that some of the iller patients had a pre-renal cause such as salt and water depletion.

The analysis shows that the patients at risk with this regimen can be identified and a strategy adopted to avoid infection such as reduction of the dose in the first cycle. Alternatively we recommend that, if these high risk patients receive a full dose of chemotherapy containing etoposide on the first cycle, a nadir blood count is obtained and consideration given to prophylactic antibiotics in those patients who are neutropenic after the first cycle. It is probably prudent to obtain nadir blood counts in further cycles and to adjust subsequent doses in severely neutropenic patients. Further studies on the pharmacology of oral and intravenous etoposide in these patients would be very helpful in trying to elucidate the pharmacological reasons for the toxicity.

This study was supported by a grant from the Cancer Research Campaign.

### References

ARBUCK, S.G., DOUGLAS, H.O., GOODWIN, P. *et al.* (1985). Pharmacokinetics of etoposide (VP) in patients with normal and abnormal liver function. *Proc. Am. Soc. Clin. Oncol.*, **4**, 40.

FELD, R., EVANS, W.K., DE BOER, G. *et al.* (1984). Combined modality induction therapy without maintenance chemotherapy for small cell carcinoma of the lung. *J. Clin. Oncol.*, **2**, 294.

HIRSCH, F.R., HANSEN, H.H., HANSEN, M. *et al.* (1987). The superiority of combination chemotherapy including etoposide based on *in vivo* cycle analysis in the treatment of small-cell lung cancer: a randomised trial of 288 consecutive patients. *J. Clin. Oncol.*, **5**, 585.

JUMA, F.D. (1984). Effect of liver failure on the pharmacokinetics cyclophosphamide. *Eur. J. Clin. Pharmacol.*, **26**, 591.

MEDICAL RESEARCH COUNCIL LUNG CANCER WORKING PARTY (1989). Controlled trial of 12 versus 6 courses of chemotherapy in the treatment of small cell lung cancer. *Br. J. Cancer* (in press).

OSTERLIND, K., SORENSON, S., HANSEN, H.H. *et al.* (1983). Continuous versus alternating combination chemotherapy for advanced small cell carcinoma of the lung. *Cancer Res.*, **43**, 6085.

PFLUGER, K.-H., SCHMIDT, L., MERKEL, M. *et al.* (1987). Drug monitoring of etoposide (VP16-213). Correlation of pharmacokinetic parameters to clinical and biochemical data from patients receiving etoposide. *Cancer Chemother. Pharmacol.*, **20**, 59.

SESSA, C., ROSSI, C., ZUCCHETTI, M. *et al.* (1985). Pharmacokinetics of VP16 in patients with impaired renal function. *Proc. Am. Assoc. Cancer Res.*, **26**, A604.

SOUHAMI, R.L., GEDDES, D.M., SPIRO, S.G. *et al.* (1984). Radiotherapy in small cell lung cancer: a randomised trial. *Br. Med. J.*, **288**, 1643.

SOUHAMI, R.L., BRADBURY, I., GEDDES, D.M. *et al.* (1985). The prognostic significance of laboratory parameters measured at diagnosis in small cell carcinoma of the lung. *Cancer Res.*, **45**, 2878.

SPIRO, S.G., SOUHAMI, R.L., GEDDES, D.M. *et al.* (1989). Duration of chemotherapy in small cell lung cancer: a Cancer Research Campaign trial. *Br. J. Cancer* (in press).

VAN DEN BERG, H.W., DESAI, Z.R., WILSON, R. *et al.* (1982). The pharmacokinetics of vincristine in man: reduced drug clearance associated with raised serum alkaline phosphatase and dose-limited elimination. *Cancer Chemother. Pharmacol.*, **8**, 215.